# Carbon and Oxygen Isotope Records from *Tridacna derasa* Shells: Toward Establishing a Reliable Proxy for Sea Surface Environments

**DOI:** 10.1371/journal.pone.0157659

**Published:** 2016-06-21

**Authors:** Junpei Yamanashi, Hideko Takayanagi, Ayaka Isaji, Ryuji Asami, Yasufumi Iryu

**Affiliations:** 1 Institute of Geology and Paleontology, Graduate School of Science, Tohoku University, Aobayama, Sendai, Japan; 2 Department of Earth & Planetary Sciences, Graduate School of Environmental Studies, Nagoya University, Furo-cho, Chikusa-ku, Nagoya, Japan; 3 Department of Earth Science, Faculty of Science, University of the Ryukyus, 1 Senbaru, Nishihara, Okinawa, Japan; University of California, UNITED STATES

## Abstract

We report the carbon (δ^13^C) and oxygen (δ^18^O) isotope records of three modern *Tridacna derasa* shells from Ishigaki-jima, southwestern Japan. The high-resolution δ^13^C profiles of samples from the inner shell layer on cross-sections fall within similar narrow ranges and display no regular variations or trends, such as an ontogenetic trend or abrupt short-term drops likely to be related to reproductive activity. This suggests that the calcification site of this species is not likely affected by photosynthetic CO_2_ uptake or CO_2_ incorporation during respiration. The δ^18^O profiles show distinct seasonal cycles. The intraspecific variability in the δ^18^O values is small in parts of the shell precipitated in the adult stage, but is not negligible in the juvenile and senescent stages. The differences in the monthly and seasonally resolved δ^18^O values among shells are less than 0.51‰ and 0.76‰, respectively. The shell δ^18^O values are nearly identical or close to the δ^18^O values for aragonite precipitated in oxygen isotope equilibrium with ambient seawater (δ^18^O_EA_). The largest differences between the shell δ^18^O and δ^18^O_EA_ values calculated from the monthly and seasonally resolved data correspond to an overestimate of the seawater temperature by as much as 1.7°C and 2.3°C, respectively. However, these differences are smaller in the adult stage (<0.25‰) than in the other stages. This small difference allows an accurate reconstruction of the seawater temperature with an error of <1.1°C. Consequently, we recommend that multiple shell records be obtained because of the non-negligible intraspecific variations in their δ^18^O values. Growth banding, composed of alternating narrow white bands and wide light-grey bands, is discernible on cross-sections of the inner shell layer. The δ^18^O_shell_ data indicate that they were formed in winter and the other seasons, respectively.

## Introduction

High-resolution paleoenvironmental records are required from various localities over the globe to understand past climate dynamics and predict future climate change. Marine carbonate-secreting organisms, such as corals and mollusks, are sensitive to the ambient environment and preserve various types of environmental information in their skeleton in the form of physical (e.g., increment width) or geochemical variations [1–3]. Of these records, the oxygen isotope ratios (δ^18^O) of biogenic carbonates has widely been used to reconstruct paleoenvironments because it commonly reflects both sea-surface temperatures (SSTs) and the δ^18^O values of the ambient seawater (δ^18^O_sw_) in which the carbonates were secreted [4–7]. Numerous paleoenvironmental studies have investigated the biogenic carbonates of foraminifers [8, 9], corals [10–14], mollusks [15–19], and brachiopods [20–24].

The tridacnids (Subfamily Tridacninae Lamarck, 1819 [25]) are some of the largest bivalves, with shell length being up to 1 m at a maximum, in geological history and have been a prominent member of Indo-Pacific coral reef communities since the Eocene [26]. All tridacnids live in the euphotic zone and are associated with unicellular algal symbionts (zooxanthellae). This association gives rise to unusually high calcification rates, attributed to light-enhanced calcification [27, 28]. The tridacnids form dense aragonitic shells with annual lines and daily growth bands in their inner shell layer [29, 30], which allow the reconstruction of paleoenvironmental change, even on a subdaily time scale [31]. The isotopic data collected previously indicate that tridacnids precipitate their shells in oxygen isotopie equilibrium with seawater [32, 33]. Patzöld et al. (1991) [34] showed that the biogenic (daily growth banding) and geochemical (δ^18^O values) records in the inner shell layer are more suitable for paleoenvironmental reconstruction than those of the outer shell layers or hinge, so the former have been used for paleoenvironmental studies [2, 30, 35–38]. Therefore, we also studied the isotopic records in the inner shell layer.

**Fig 1:**
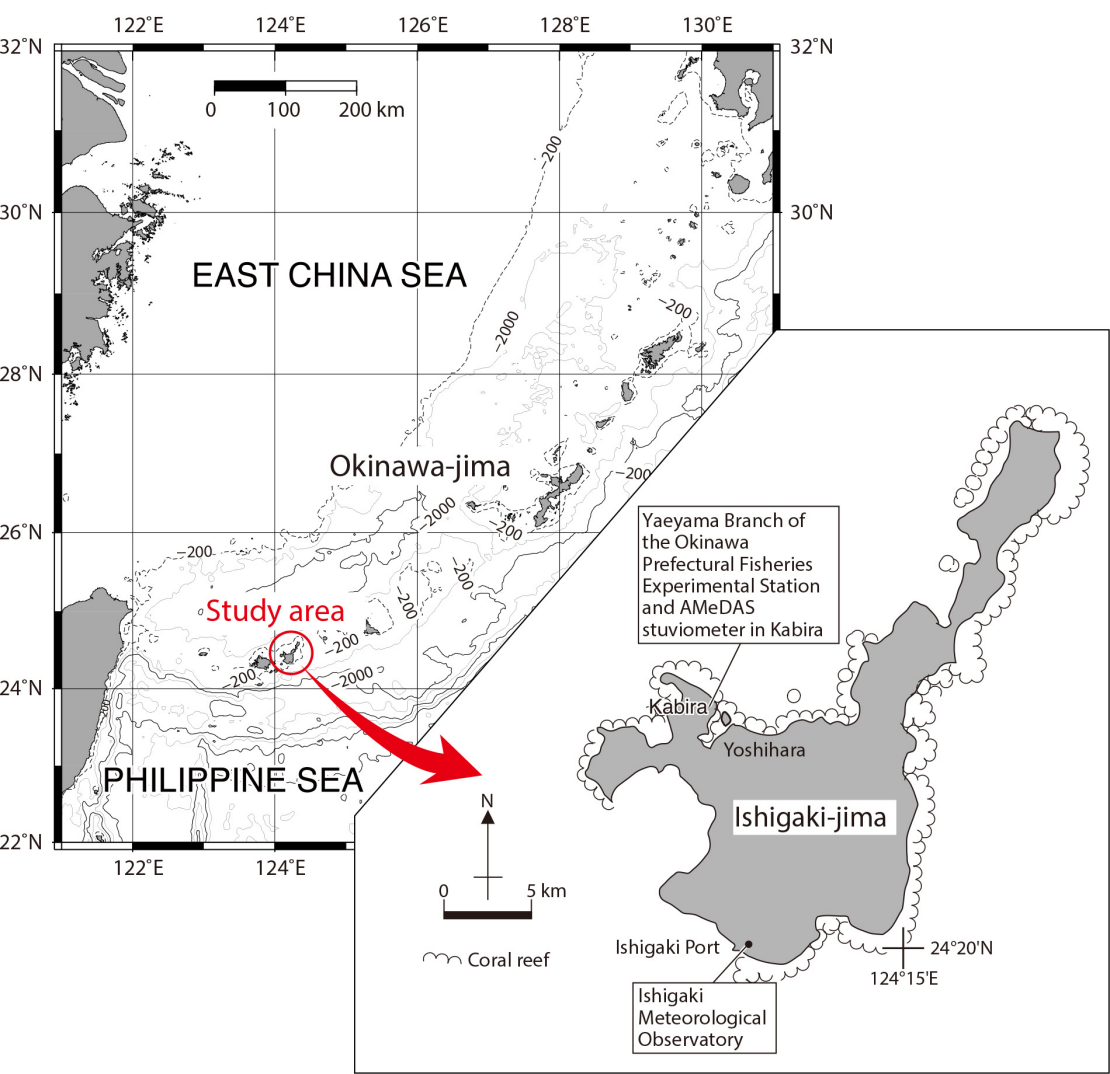
Location and map of the study site. (A) Study area in Ishigaki-jima (Ryukyu Islands). Bathymetric data are from ETOPO1 Global Model [40]. (B) Map of Ishigaki-jima.

**Fig 2:**
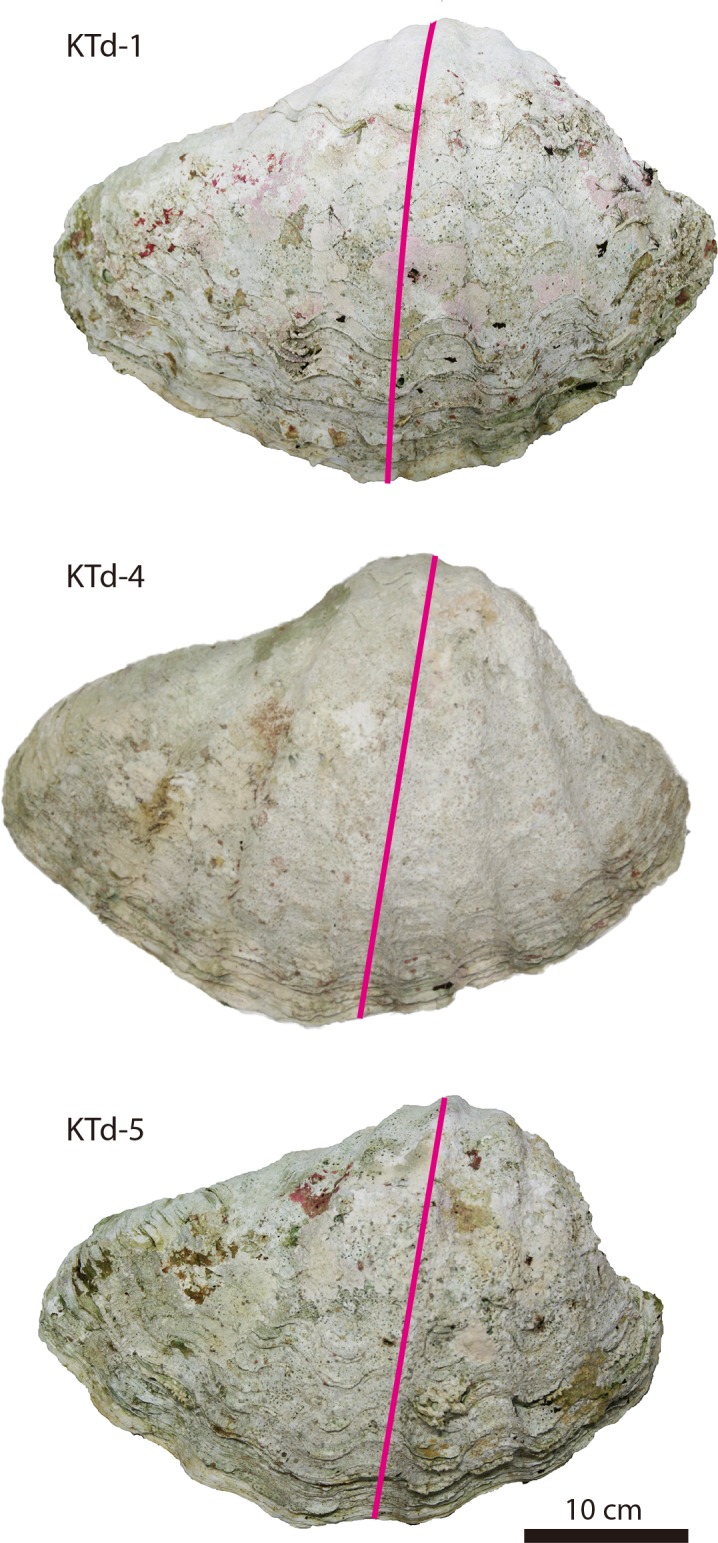
Photographs of the studied *Tridacna derasa* shells. The shells (left valves) were cut vertically (pink lines) along their maximum growth axes.

**Fig 3:**
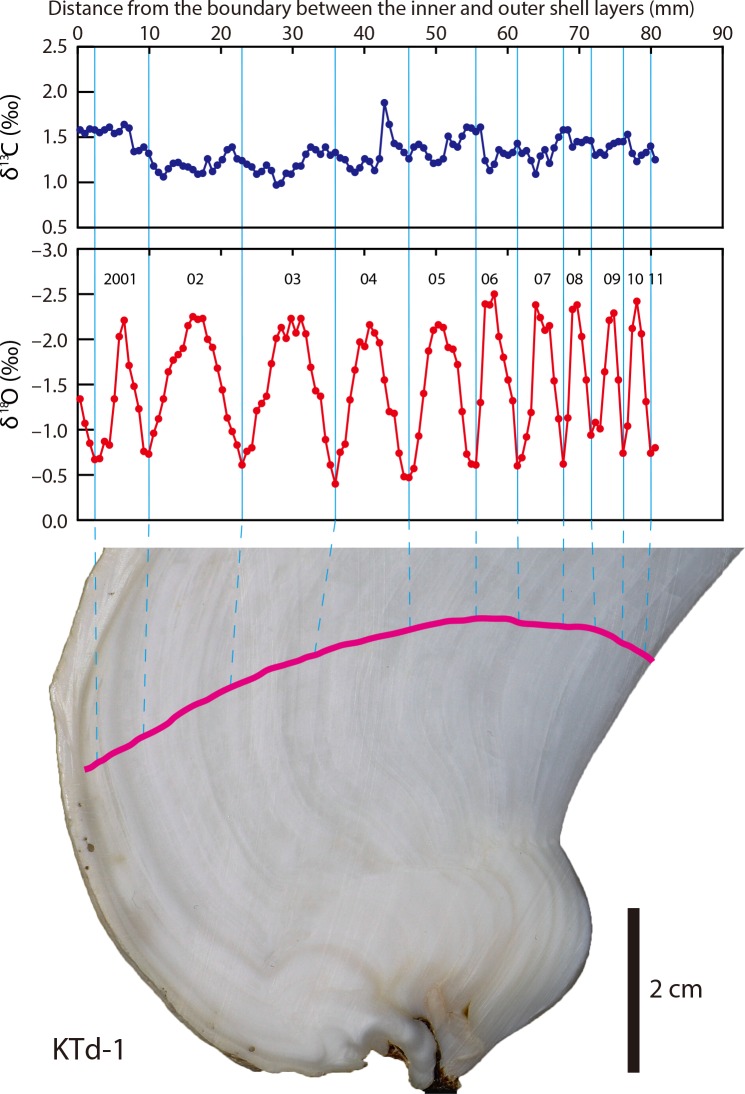
δ^13^C_shell_ and δ^18^O_shell_ profiles of a *Tridacna derasa* shell (KTd-1). Pink line indicates the sampling transect.

**Fig 4:**
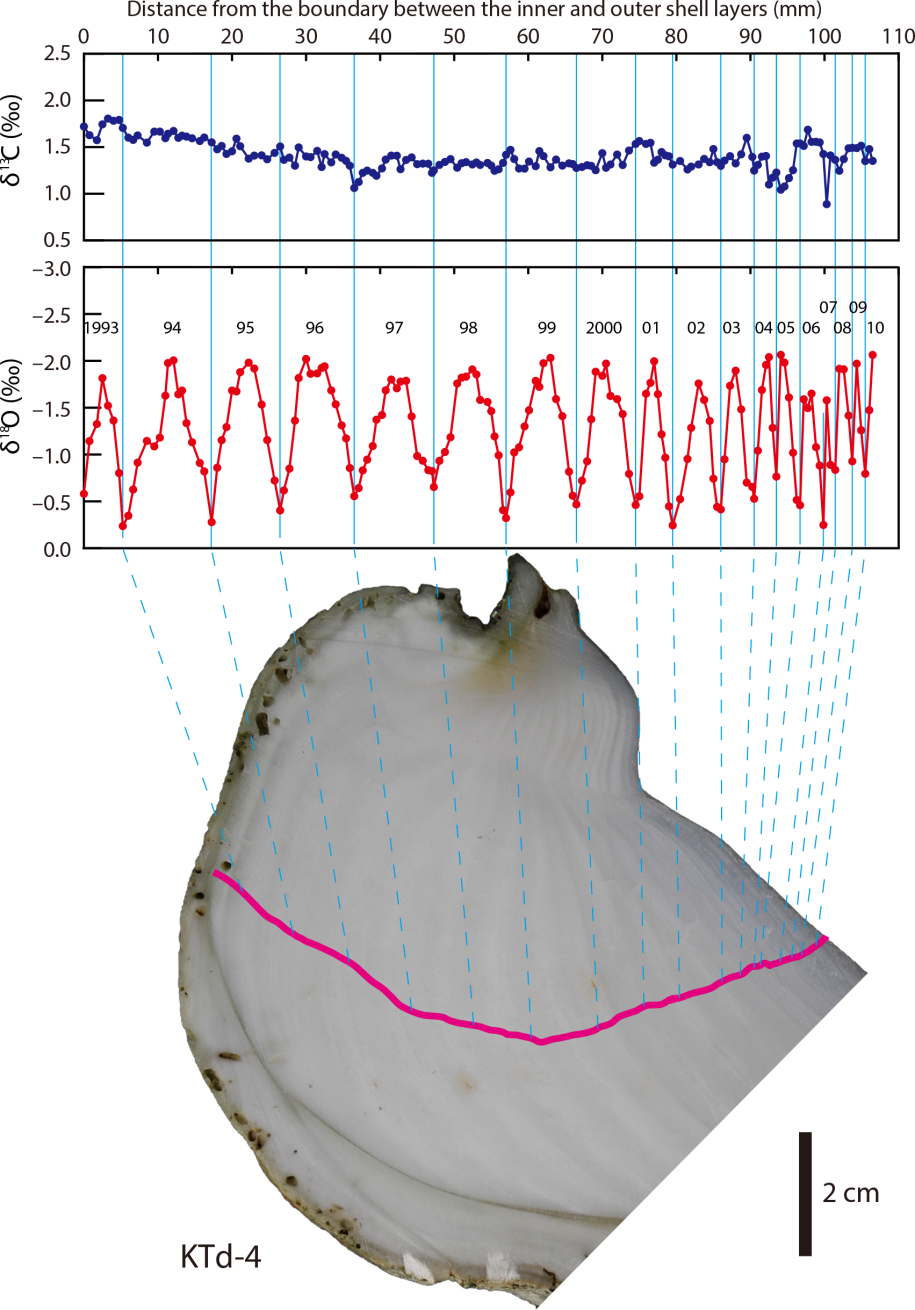
δ^13^C_shell_ and δ^18^O_shell_ profiles of a *Tridacna derasa* shell (KTd-4). Pink line indicates the sampling transect.

**Fig 5:**
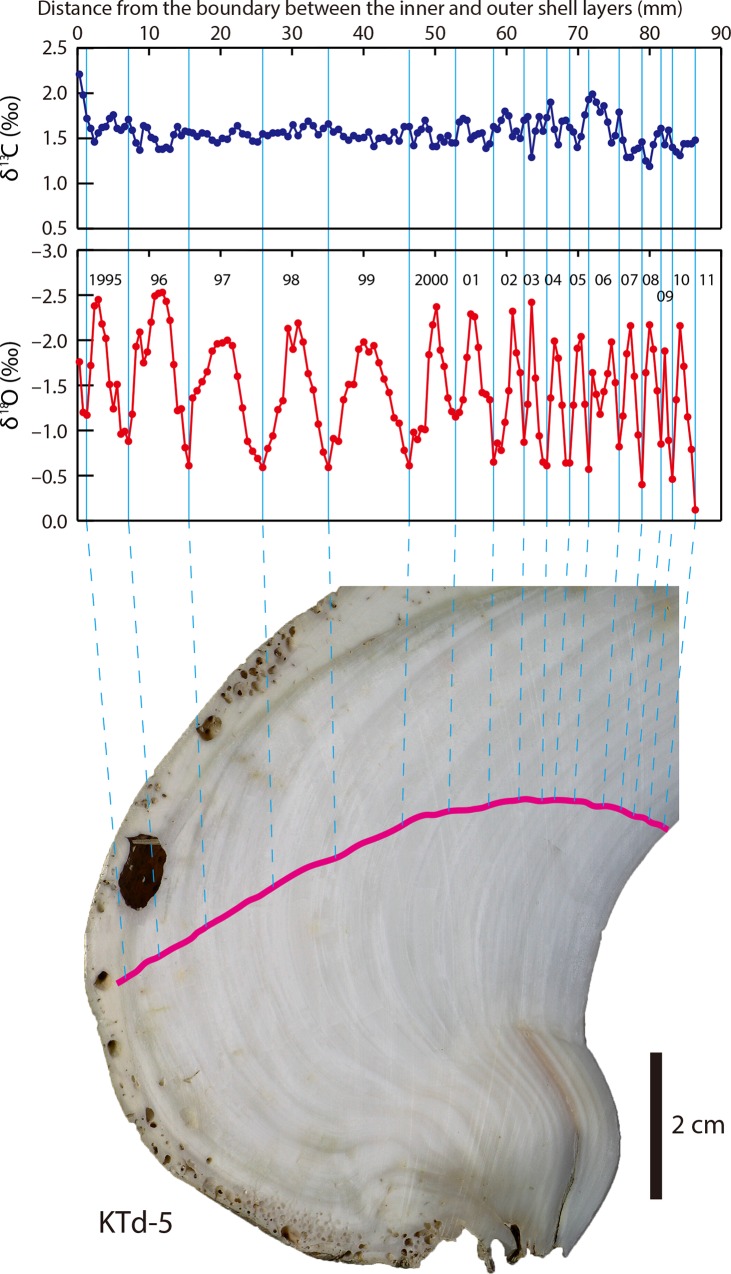
δ^13^C_shell_ and δ^18^O_shell_ profiles of a *Tridacna derasa* shell (KTd-5). Pink line indicates the sampling transect.

**Fig 6:**
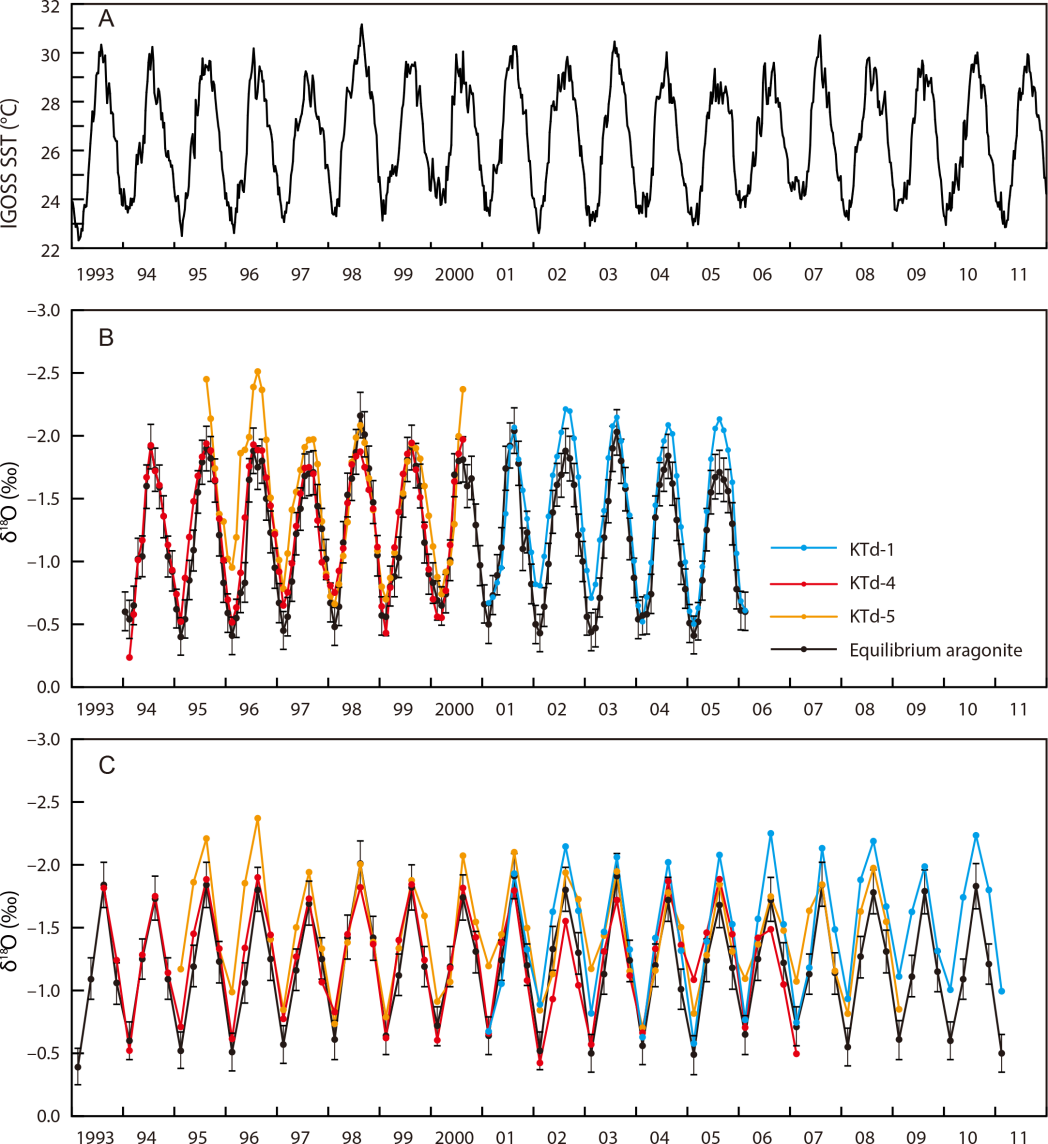
Comparison of time series of sea surface temperature and δ^18^O_shell_ values for *Tridacna derasa* shells. (A) IGOSS SST data around Ishigakiji-jima (1° resolution gridded data, centered at 24°30′N and 124°30′E) during the period 1993–2011. (B) Monthly resolved δ^18^O_**shell**_ profiles of the KTd-1, KTd-4, and KTd-5 shells and equilibrium aragonite. (C) Seasonally resolved δ^18^O_shell_ profiles of the KTd-1, KTd-4, and KTd-5 shells and equilibrium aragonite. Each error bars indicates ±1 standard error.

However, some issues remain to be resolved when using tridacnid δ^18^O values as reliable paleoenvironmental proxies. One of the most critical issues is that previous studies of tridacnid δ^18^O values predominantly dealt with isotopic data from a single shell of a single taxon, so neither the inter- nor intraspecific variations were fully considered [2, 30, 35–37]. In this article, we first report the intraspecific (= intershell) variations in carbon (δ^13^C_shell_) and oxygen (δ^18^O_shell_) isotope ratios of samples from the inner shell layer on cross-sections of modern *Tridacna derasa* (Röding, 1798) [39]. The studied materials were three shells collected from Ishigaki-jima, Ryukyu Islands, southwestern Japan (Figs 1 and 2) [40]. We then compare the δ^18^O_shell_ profiles of this species with each other and with oceanographic data around Ishigaki-jima (Figs 3–6). In this way, we show the extent to which the δ^18^O_shell_ values of *T*. *derasa* reliably record oceanographic conditions, especially SSTs.

## Materials and Methods

### Study site and climate regime

The Ryukyu Islands are located to the southwest of mainland Japan and are composed of several tens of islands and islets (Fig 1A). These islands are arranged in a curved row, known as the Ryukyu Island Arc. Most of the islands are rimmed by well-developed fringing coral reefs, although they are located near the northern limit of the coral reef province in present-day oceans.

Ishigaki-jima is located in the southwestern part of the Ryukyus (24°19′–37′N, 124°4′–21′E; Fig 1A). The climate on the island is subtropical (Table 1). The monthly mean atmospheric temperature measured at the Ishigaki Meteorological Observatory ranges measured at from 18.6°C (January) to 29.5°C (July), with an annual mean of 24.3°C. Semidiurnal tides are clearly evident throughout the islands, with a maximal range of 1.9 m at spring tide and 1.0 m at neap tide. The annual rainfall reaches 2100 mm, with rainy months in May–June and August–October. The prevailing wind is SSE in summer and NNW in winter. A monitoring record from the sea surface at Ishigaki Port during 1998–2004 showed an average sea surface salinity (SSS) of 34.3, generally ranging from 33.6 to 35.0 and associated with short-term drops of <2 caused by heavy rainfall [41]. The Integrated Global Ocean Services System (IGOSS) data (1° resolution gridded data, centered at 24°30′N and 124°30′E) for 1993–2011 suggest that the SSTs varied between 22.3°C and 31.2°C, with an annual mean of 26.5°C. The highest and lowest monthly mean SSTs were 30.6°C in August and 22.6°C in February, respectively [42].

**Table 1:** Climate in Ishigaki-jima.

	Atmospheric temperature (°C)^1^	Sea surface temperature (°C)^2^	Rainfall (mm)^1^
Annual mean	24.3	26.4	2106.8
Mean monthly maximum	29.5 (July)	30.6 (August)	261.6 (August)
Mean monthly minimum	18.6 (January)	23.1 (February)	126.3 (December)

1 Data for 1981–2010 are from the Japan Meteorological Agency

2 Integrated Global Ocean Services System (IGOSS) data (1° resolution gridded data, centered at 24°30′N and 124°30′E) for 1994–2011.

### Tridacna derasa

There are currently eight described species within the genus *Tridacna* Bruguière, 1797 [43, 44]. They are among the most conspicuous marine invertebrates on coral reefs due to their large size and brilliantly colored mantle that contains photosynthesizing symbionts [44]. Of these species, *T*. *derasa* shells were examined in this study. *Tridacna derasa*, *T*. *gigas*, *T*. *crocea*, *T*. *squamosa*, and *T*. *maxima* are widely distributed in the Indian and Pacific Oceans, with the latter two extending their distribution into the Red Sea, whereas *T*. *squamosina*, *T*. *rosewateri*, and *T*. *mbalavuana* have restricted distributions (Red Sea, Mauritius, and Fiji to Tonga, respectively) [44]. Shell length of *T*. *derasa* reaches 50–60 cm. They live byssally anchored at a juvenile stage, but when approaching maturity their byssus glands atrophy, and the adult clams take up unattached existence on coral rubble or hard packed sand [45]. Phylogenetic analyses indicated that the divergent time was estimated to be ~10 Ma between *T*. *derasa* and *T*. *gigas*-*T*. *mbalavuana* [44].

### Materials

Three *T*. *derasa* shells, designated KTd-1, KTd-4, and KTd-5 (Fig 2), were collected in 1990 (KTd-4 and KTd-5) and 1998 (KTd-1) at the stage of fertilized egg and grown at a depth of 2 m in a large culture pond at the Yaeyama Branch of the Okinawa Prefectural Fisheries Research and Extension Center located at Kabira, Ishigaki-jima, Ryukyu Islands, southwestern Japan (24°28′N, 124°09′E; Fig 1B). They were collected on 7 May 2011 (KTd-4 and KTd-5) and 12 May 2011 (KTd-1). The water temperature and salinity in the pond were roughly equivalent to those of the outer ocean because the seawater was always pumped up from the outer ocean into the pond. The shell height and length of KTd-1 were 25.8 cm and 40.4 cm, respectively, those of KTd-4 were 26.6 cm and 41.7 cm, respectively, and those of KTd-5 were 25.3 cm and 38.3 cm, respectively. After the soft tissue was removed, a ~1 cm thick slab was cut vertically from each shell along the maximum growth axis (Fig 2). The inner and outer shell layers were clearly discernible on each slab. Carbonate samples for isotope analysis were manually obtained along a roughly median line on the inner shell layer at ~0.5–0.7 mm intervals using drill bits with diameters of 0.6 mm and 1.0 mm (Figs 3–5). The numbers of carbonate samples obtained from KTd-1, KTd-4, and KTd-5 were 121, 163, and 149, respectively.

### Isotope analysis

Stable isotope analyses of the shell aragonite were performed with a Thermo Scientific DeltaV Advantage mass spectrometer, coupled with a ThermoQuest Kiel III Carbonate Device, at the Graduate School of Science, Tohoku University, Sendai, Japan. The δ^13^C_shell_ and δ^18^O_shell_ values were calibrated for the NBS-19 international standard relative to VPDB. The external precision (1σ) for the δ^13^C and δ^18^O analyses, based on replicate measurements of the laboratory reference sample (JCt-1; [46]), were ±0.02‰ and ±0.04‰ (*n* = 112), respectively. The correlations between the δ^13^C_shell_ and δ^18^O_shell_ values were evaluated with a reduced major axis regression technique [47], the significance of which was examined statistically with a two-sided Pearson test and a 95% confidence limit.

The distance domain δ^18^O_shell_ profiles clearly showing seasonal cycles were converted to time series for better interpretation and comparison with those of aragonite precipitated in oxygen isotope equilibrium with ambient seawater (equilibrium aragonite), because it is well known that the aragonitic shells of tridacnids are generally precipitated in oxygen isotope equilibrium with ambient seawater [30, 32]. The temporal resolution of the δ^18^O_shell_ values, represented by the number of isotope data per year, varied from ~19 days to ~4 months per sample. Therefore, we converted the distance domain δ^18^O_shell_ profiles to time series with peak-to-peak matching (i.e., annual maximum and minimum values in a year) with the equilibrium aragonite δ^18^O profiles using AnalySeries software [48]. The time for the data point of annual maximum and minimum δ^18^O_shell_ values were assigned to February and August, respectively. The monthly and seasonally resolved shell δ^18^O profiles were then calculated on the assumption of constant growth between each shell δ^18^O peak. The δ^13^C_shell_ time series were generated simultaneously.

## Results

### δ^13^C_shell_ and δ^18^O_shell_ values

The δ^13^C_shell_ profiles of KTd-1, KTd-4, and KTd-5 showed no distinct cycles (Figs 3–5). The δ^13^C_shell_ values for KTd-1, KTd-4, and KTd-5 ranged from 0.97‰ to 1.88‰ (average = 1.33‰, σ (standard deviation) = 0.17‰), from 0.89‰ to 1.81‰ (average = 1.40‰, σ = 0.15‰), and from 1.19‰ to 2.21‰ (average = 1.56‰, σ = 0.15‰), respectively (S1 Table).

The δ^18^O_shell_ profiles are characterized by a series of regular cycles of varying amplitudes and frequencies. The numbers of cycles in the profiles of KTd-1, KTd-4, and KTd-5 are 10, 17, and 16, respectively (Figs 3–5). The δ^18^O_shell_ values for KTd-1, KTd-4, and KTd-5 range from –2.50‰ to –0.40‰ (average = –1.47‰, σ = 0.59‰), from –2.06‰ to –0.24‰ (average = –1.26‰, σ = 0.51‰), and from –2.53‰ to –0.12‰ (average = –1.46‰, σ = 0.54‰), respectively (S1 Table).

### δ^13^C_EA_ and δ^18^O_EA_ values

We estimated the approximate ranges of the δ^13^C and δ^18^O values of equilibrium aragonite (δ^13^C_EA_ and δ^18^O_EA_, respectively) using previously published δ^13^C values of dissolved inorganic carbon (δ^13^C_DIC_) and δ^18^O_sw_, respectively, of seawater samples collected around Okinawa-jima [23] and Ishigaki-jima [41]. The δ^13^C_DIC_ values at Kabira, where *T*. *derasa* grew, were assumed to be 1.1‰–1.6‰. Because the pH of the surface seawater at Ishigaki-jima ranged from 7.9 to 8.0, it was assumed that the δ^13^C_HCO3_^_^ values for this seawater were ~0.2‰ greater than the δ^13^C_DIC_ values [49–51]. Therefore, the δ^13^C_EA_ values calculated using the δ^13^C_DIC_ values (1.1‰–1.6‰) and the aragonite HCO_3_^–^-enrichment factor (2.7 ± 0.6‰; [50]) should range from 3.4‰ to 5.1‰ at the *T*. *derasa* growth site.

The δ^18^O_EA_ time series were calculated using the IGOSS SST data [42] and the monthly average δ^18^O_sw_ values at Ishigaki Port, ~14 km south of the *T*. *derasa* growth site, during the period from December 1997 to May 2004, measured by Abe et al. (2009) [41] and based on the following equation [52]:

$$10^{3}{ln\alpha }_{aragonite-water}=(18.45\pm 0.4)*10^{3}/T(K)-(32.45\pm 1.5)$$

The δ^18^O_sw_ values [41] varied between 0.09‰ and 0.29‰, and showed clear seasonal cycles, except for abrupt short-term drops (down to –0.30‰) during or just after heavy rainfall. The monthly average δ^18^O_EA_ values were calculated, and range from –0.31‰ to –2.16‰ (annual mean of –1.18‰,), which demonstrated distinct seasonal cycles (Fig 6). The annual maximum and minimum δ^18^O_EA_ values were recorded in August and February, respectively.

## Discussion

### δ^13^C_shell_ values

Because *T*. *derasa* is a zooxanthellae (symbiont)-bearing giant clam, its metabolic activity is expected to be closely related to organic carbon production by zooxanthellan photosynthesis, which is considered to show seasonal cycles corresponding to those of solar radiation. However, the δ^13^C_shell_ values show no seasonal cycles (Figs 3–5). This is true for the inner shell layers of not only *T*. *derasa* but also other tridacnids [35, 38, 53–55]. These indicates that such a link is unlikely because only minor amounts of metabolic carbon are incorporated into the bivalve shells as shown in non-zooxanthellate bivalves [56, 57].

The δ^13^C_shell_ values show no statistically significant ontogenetic trend. Previous studies have shown the same results in the inner shell layers of other tridacnids [32, 35, 38, 55]. In contrast, distinct ontogenetic decreases in δ^13^C values have been detected in some bivalves [58–60]. Based on the relationship between metabolic rate and body size, Lorrain et al. (2004) [60] attributed this ontogenetic decrease to the increased incorporation of respiratory CO_2_ during growth. As mollusks grow, more metabolic (= respiration-derived) CO_2_ becomes available to them, whereas the amount required for shell formation decreases, resulting in the incorporation of more metabolic carbon (^12^C-enriched) into their shells. However, ontogenetic increases in δ^13^C values are known from non-zooxanthellate and zooxanthellate (tridacnid) bivalve shells, indicating that the model proposed by Lorrain et al. (2004) [60] may not be a general model for all bivalves [19, 53].

The spawning period *Mytilus edulis* is reflected by more negative δ^13^C_shell_ values, although the δ^13^C_DIC_ is generally becoming more positive, which is explained by higher metabolic rates just after spawning, as energy lost during spawning is restored [61]. Vander Putten et al. (2000) [62] also reported these patterns in δ^13^C_shell_ values in *M*. *edulis* as being a result of increased respiration associated with periods of higher food availability. However, no such negative peaks have been found in the δ^13^C profiles of *T*. *derasa* or other tridacnid shells [32, 35, 38, 55].

It was shown that decadal variability of δ^13^C_DIC_ values relating to phytoplankton productivity and large-scale ocean dynamics are possible causes of ontogenetic trends of δ^13^C values from long-lived bivalve shells [63]. However, the three δ^13^C_shell_ profiles of *T*. *derasa* are not long enough to discuss such relationships.

**Fig 7:**
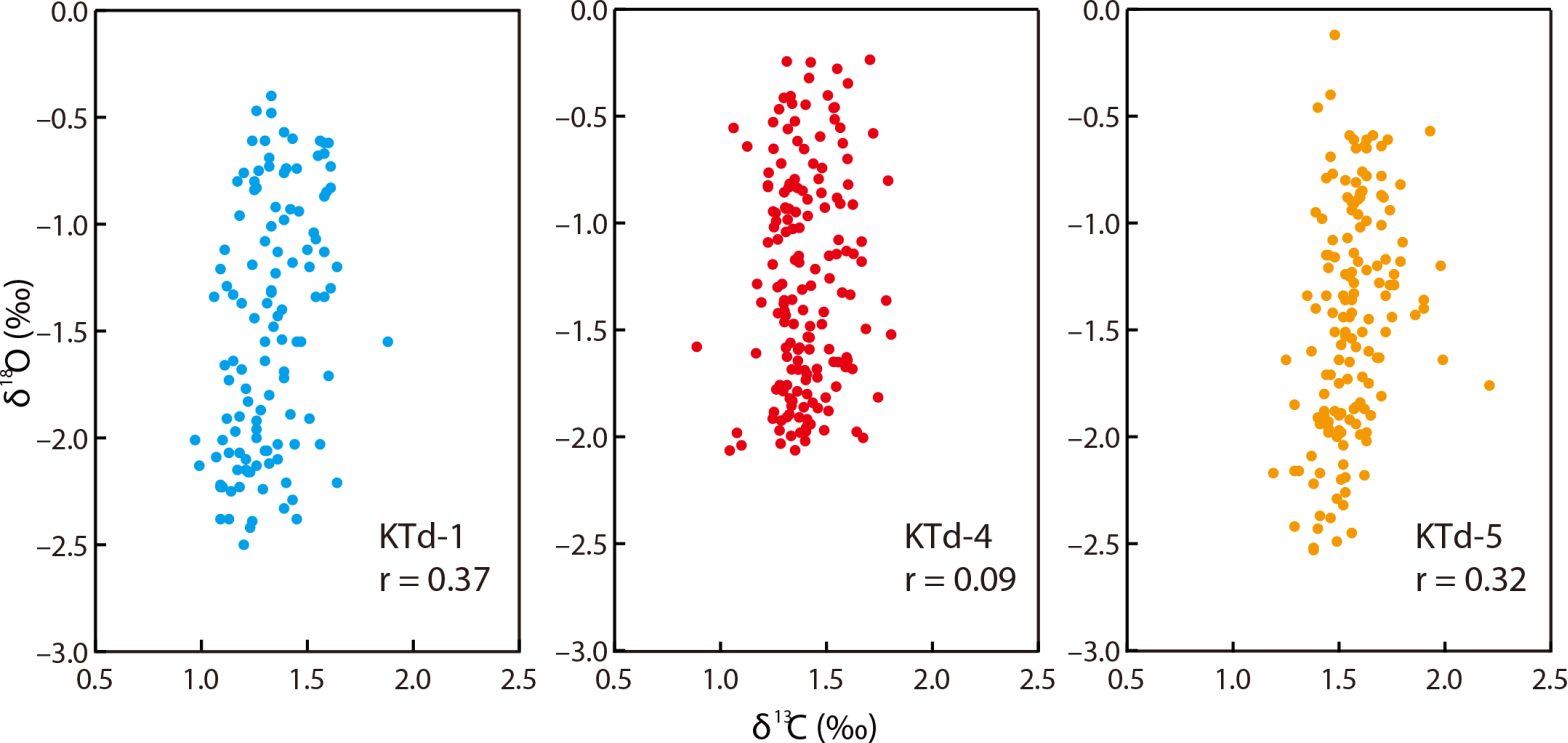
Cross-plots of δ^13^C_shell_ versus δ^18^O_shell_ for the studied *Tridacna derasa* shells. The δ^13^C_shell_ and δ^18^O_shell_ values show weak or no significant correlations.

The relatively constant δ^13^C_shell_ values, characterized by the absence of seasonal cycles, ontogenetic decreases, and abrupt short-term drops in δ^13^C_shell_ that are attributable to reproductive activity, suggests that the calcification site of this species is not affected by CO_2_ uptake resulting from photosynthesis or the incorporation of CO_2_ from respiration. This is common in other tridacnid species [32, 35, 38, 55]. The δ^13^C_shell_ and δ^18^O_shell_ values show weak or no significant correlations (KTd-1, *r* = 0.37, *p* < 0.05; KTd-4, *r* = 0.09, *p* < 0.05; KTd-5, *r* = 0.32, *p* < 0.05; Fig 7), suggesting that there is no kinetic effect [64] or a very weak one on isotope fractionation during the precipitation of the carbonate-forming *T*. *derasa* shells.

The δ^13^C_shell_ values are 1.0‰–2.2‰ lower than the lowest δ^13^C_EA_ values (3.4‰). At present, we have no explanation why the δ^13^C_shell_ values are so low relative to the δ^13^C_EA_ values. Possible causative factors include the pH at the calcification site, as pH is known to affect the δ^13^C and δ^18^O values of skeletal carbonates [51, 56, 65].

### δ^18^O values

The δ^18^O_shell_ profiles are characterized by a series of regular seasonal cycles of varying amplitudes and frequencies. The amplitude of the cycles in the δ^18^O_shell_ profiles ranges from 1.03‰ to 1.90‰ for KTd-1, from 1.19‰ to 1.73‰ for KTd-4, and from 1.16‰ to 2.04‰ for KTd-5. Because the seasonal amplitude of the δ^18^O_sw_ values is <0.2‰ (excluding the extremely low outliers recorded during or just after short-term heavy rainfall), the contribution of δ^18^O_sw_ to the δ^18^O_shell_ variations is less than <19%. Therefore, the cycles of the δ^18^O_shell_ profiles correspond predominantly to seasonal changes in seawater temperature. δ^18^O_shell_ profiles characterized by distinct seasonal cycles have previously been reported for the inner shell layers of tridacnids [30, 33–35, 37, 53, 54, 66].

**Fig 8:**
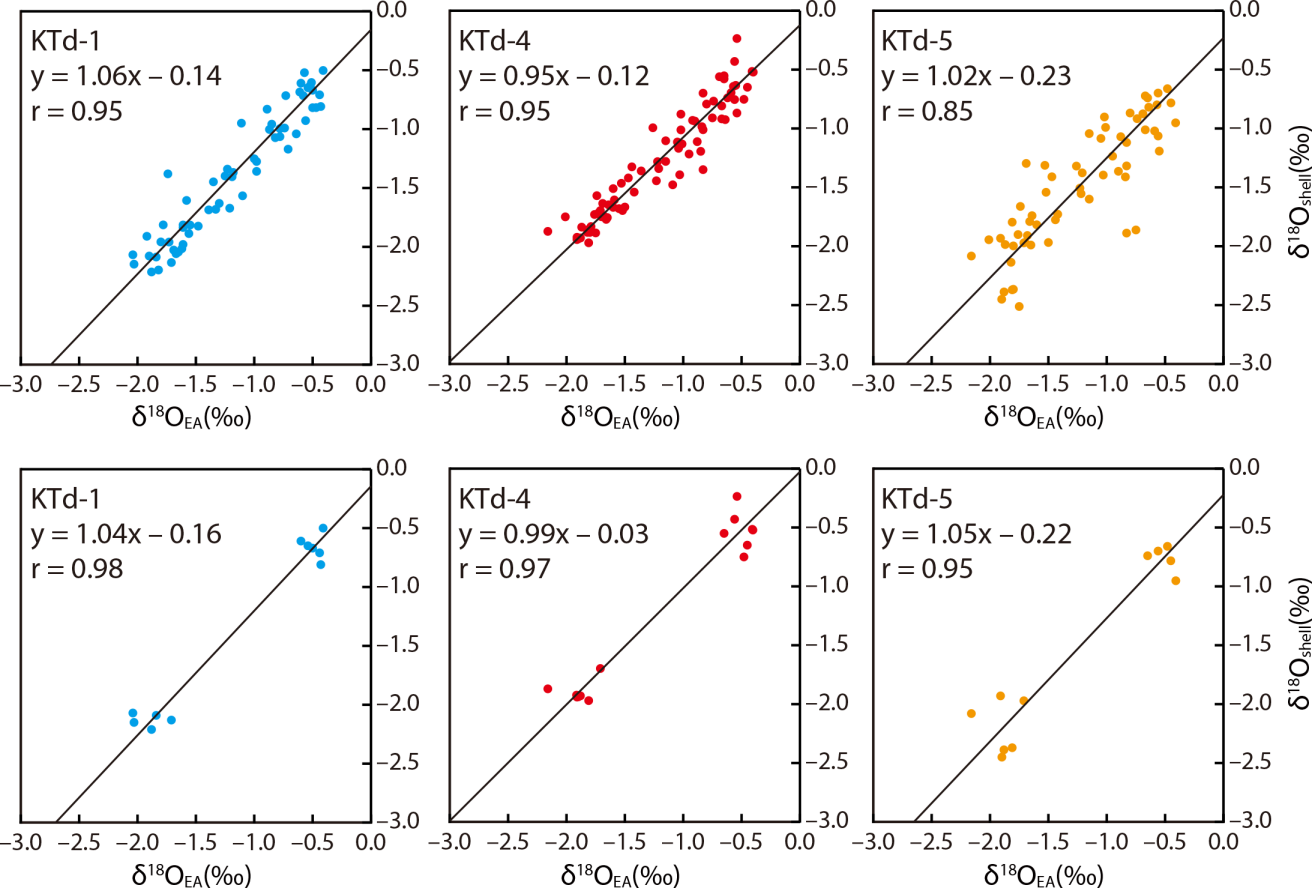
Cross-plots of monthly resolved δ^18^O_shell_ versus δ^18^O_EA_ for the studied *Tridacna derasa* shells. All monthly resolved data are shown in the upper row. Annual maximum and minimum δ^18^O_shell_ values (= δ^18^O_shell_ values for the coolest and warmest months, respectively) are indicated in the lower row. Black line is the regression line.

The aragonitic shells of tridacnids are known to be precipitated in oxygen isotope equilibrium with ambient seawater [30, 32]. However, the Δδ^18^O values, defined as the δ^18^O_shell_ values minus the δ^18^O_EA_ values, are not negligible, ranging from –0.25‰ to 0.00‰ (KTd-1; *n* = 11), from –0.12‰ to 0.15‰ (KTd-4; *n* = 14), and from –0.38‰ to 0.00‰ (KTd-5; *n* = 11), if we calculate them using the δ^18^O_shell_ and δ^18^O_EA_ values for the coolest and warmest months in the monthly resolved data from the shell portion in which 12 or more samples for isotope analysis were collected (Fig 6B). The differences are larger if calculated from the summer and winter values using seasonally resolved data (KTd-1, –0.35‰ to 0.00‰, *n* = 21; KTd-4, –0.45‰ to 0.07‰, *n* = 28; KTd-5, –0.52‰ to 0.00‰, *n* = 29) (Fig 6C). These differences generally cause the reconstructed seawater temperatures to be overestimated. The largest monthly and seasonally resolved Δδ^18^O values correspond to differences of 1.7°C and 2.3°C, respectively. However, the monthly time series for δ^18^O_shell_ agrees well with that for δ^18^O_EA_, except for the period 1995–1997 and the warmest months in 2000 for the KTd-5 profile (Fig 6C). The small differences (<0.25‰) between δ^18^O_shell_ and δ^18^O_EA_ for 1997–2006 allow the accurate reconstruction of seawater temperatures, with an error of <1.1°C. Cross-plots of δ^18^O_shell_ versus δ^18^O_EA_ indicate that these values are not completely identical, but correlate positively, with the slopes and intercepts of the regression lines ranging from 0.95 to 1.06 and from –0.23 to –0.12, respectively, and the cross-correlation coefficients ranging from 0.73 to 0.91 (*p* < 0.05) for all monthly resolved data (Fig 8). If annual maximum and minimum δ^18^O_shell_ values (= δ^18^O_shell_ values for the coolest and warmest months, respectively) are used, the slopes and intercepts of the regression lines range from 0.99 to 1.05 and from –0.22 to –0.03, respectively, and the cross-correlation coefficients are 0.91–0.97 (*p* < 0.05) (Fig 8). Taking into account the statistical errors (δ^18^O_EA_ estimation and sampling errors), these results suggest that the seawater temperatures reconstructed from the δ^18^O_shell_ values are largely the same as the actual temperatures.

The intraspecific variability in the δ^18^O_shell_ values was relatively small in the period between 1997 and 2006 but relatively large in the periods between 1995 and 1997 (0.51‰ on monthly data and 0.46‰ on seasonal data between KTd-4 and KTd-5) and between 2006 and 2009 (0.76‰ between KTd-1 and KTd-4/5) (Fig 6). These differences may be due, at least in part, to different sampling resolutions and/or biological offsets in the juvenile and senescent stages of shell growth, which have been reported in several studies (brachiopods [23, 24, 67, 68], mollusks [69]). Therefore, the high (more than monthly) temporal resolution of the δ^18^O_shell_ values in the adult stage of shell growth are most suitable for paleoenvironmental reconstructions using *T*. *derasa*.

The shells of *T*. *derasa* (and other tridacnids) have potential advantages over the skeletal carbonates of other organisms, such as brachiopods and corals, because they are precipitated very close to oxygen isotope equilibrium with ambient seawater at least along the axis of maximum growth, which allows the δ^18^O_shell_ values from any part of the inner shell layer to be converted directly to seawater temperature if the contribution of δ^18^O_sw_ is negligible. The secondary shell layer of brachiopod shells is believed to be precipitated in carbon and oxygen isotope equilibrium with ambient seawater. However, recent investigations have shown that the δ^13^C and δ^18^O values of modern brachiopod shells may be partly or wholly outside the range of those values for equilibrium calcite, which is attributed, at least in part, to kinetic and metabolic isotope fractionation effects [22–24, 67, 68, 70–73] or unidentified chemical conditions at the calcification sites [23]. It is well known that the δ^13^C and δ^18^O values of coral skeletons deviate significantly from those of equilibrium aragonite because of the effects of kinetic and metabolic isotope fractionation [64]. Our study shows that the δ^18^O_shell_ values for the adult growth stages of *T*. *derasa* shells, with little intraspecific variability, are in good agreement with the δ^18^O_EA_ values. This relationship can be used to generate high-resolution δ^18^O_shell_ time series of seawater temperatures and δ^18^O_sw_ in coral reef environments. It should also be noted that the use of multiple shell samples provides a more reliable reconstruction of seawater temperatures, with an error of <1.1°C.

### Growth curves and growth lines

**Fig 9:**
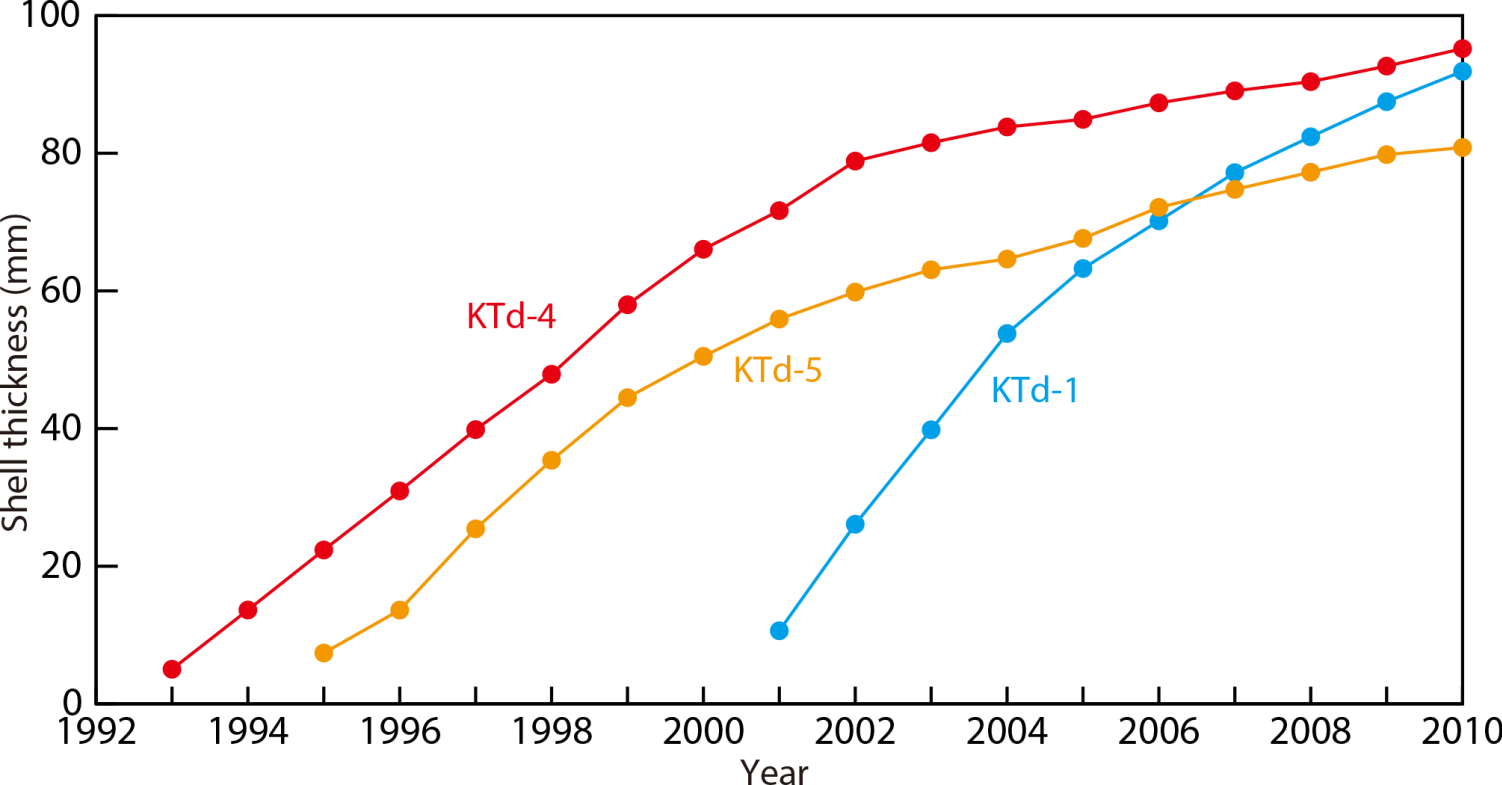
Annual rate of shell thickening of *Tridacna derasa* estimated from seasonal variations in δ^18^O_shell_ values. Each annual increment represents a distance between annual δ^18^O_shell_ maxima, which was measured perpendicular to growth lines/bands.

Seasonal variations in δ^18^O_shell_ allowed the construction of growth curves for the tridacnid shells studied. A comparison of the δ^18^O_shell_ and δ^18^O_EA_ profiles indicated life spans of 10, 17, and 16 years for KTd-1, KTd-4, and KTd-5, respectively (Fig 6). The annual rate of shell thickening defined as a distance between annual δ^18^O_shell_ maxima (= seawater temperature minima), which was measured perpendicular to growth lines/bands, was 5.0–15.5 mm/year during the juvenile to adult stages, and decreased to 1.0–7.2 mm/year during the senescent stage. The growth curves representing shell thickening (Fig 9) show similar shapes to those of many other organisms characterized by growth rates that are initially high and later low (tridacnids [32, 33, 74], bivalves [75], brachiopods [72]).

Clear growth banding, composed of alternating wide light-grey bands and narrow white bands, is discernible on cross-sections of the inner shell layer (Figs 3–5). Different terms have been used to describe the growth bands, depending partly on differences in the instruments used to observe them, such as transmitted [32, 36, 74] or reflected illumination [2, 55], which has led to confusion. In this study, we describe the growth bands based on observations made under reflected light. The annual δ^18^O_shell_ maxima (= seawater temperature minima) correlated with the white growth bands. This indicates that the white bands were predominantly formed during winter, which is consistent with previous findings [2, 34, 36, 74].

## Conclusions

We investigated the intraspecific variations in the δ^13^C_shell_ and δ^18^O_shell_ values of samples from the inner shell layers, taken from cross-sections, of three modern *T*. *derasa* shells from Ishigaki-jima, Ryukyu Islands, southwestern Japan. The results can be summarized as follows.

The δ^13^C_shell_ profiles of the samples fall into a relatively narrow range and show no seasonal cycles, ontogenetic decreases, or abrupt short-term drops that might be related to reproductive activity. These observations suggest that the calcification site of *T*. *derasa* is unlikely to be affected by CO_2_ uptake or influx caused by photosynthesis or respiration, respectively. The δ^13^C_shell_ values and the δ^18^O_shell_ values show no or very weak positive correlations, indicating no or little kinetic isotope fractionation during the carbonate precipitation of the *T*. *derasa* shells.

The δ^18^O_shell_ profiles are characterized by distinct cycles corresponding to seasonal changes in seawater temperature. The δ^18^O_shell_ values are usually greater than the δ^18^O_EA_ values by up to 0.38‰ and 0.52‰ when calculated from monthly and seasonally resolved data, respectively. These differences lead to seawater temperature to be overestimated by 1.7°C and 2.3°C, respectively. However, these differences are small (<0.25‰) in the parts of the shell that formed in the adult stage, which allows the reconstruction of accurate seawater temperatures with an error of <1.1°C. Therefore, the high-temporal-resolution δ^18^O_shell_ data from the adult stage are most suitable for paleoenvironmental reconstructions using *T*. *derasa*. However, the maximum intraspecific differences in the monthly and seasonally resolved δ^18^O_shell_ values are as large as 0.51‰ and 0.76‰, corresponding to differences in water temperature of 2.2°C and 3.3°C, respectively. This result suggests that multiple δ^18^O_shell_ records should be used to reconstruct seawater temperatures, because although the intraspecific variations in the δ^18^O values are not negligible, especially during the juvenile and senescent stages. If multiple data are collected, the reconstructed seawater temperatures are expected to be very close to the actual temperatures.

Cross-sections of the inner shell layer are characterized by growth banding, composed of alternating wide light-grey bands and narrow white bands. The δ^18^O_shell_ data indicate that the narrow white bands and the wide light-grey bands were formed in winter and the other seasons, respectively (Figs 3–5).

## Supporting Information

S1 Table Stable carbon and oxygen isotope ratios of the KTd-1, KTd-4, and KTd-5 shells. (XLSX)

## References

[1] Schöne BR, Fiebig J, Pfeiffer M, Gless R, Hickson J, Johnson ALA, et al Climate records from a bivalve Methuselah (*Arctica islandica*, Mollusca; Iceland). Palaeogeogr Palaeoclimatol Palaeoecol. 2005;228:130–148. 10.1016/j.palaeo.2005.03.049

[2] Aubert A, Lazareth C, Cabioch G, Boucher H, Yamada T, Iryu Y, et al The tropical giant clam *Hippous hippopus* shell, a new archive of environmental conditions as revealed by sclerochronological and δ^18^O profiles. Coral Reefs. 2009;28:989–998. 10.1007/s00338-009-0538-0

[3] Maier E, Titschack J. Spondylus ggaederopus: a new Mediterranean climate archive-based on high-resolution oxygen and carbon isotope analyses. Palaeogeogr Palaeoclimatol Palaeoecol. 2010;291:228–238. 10.1016/j.palaeo.2010.02.032

[4] Epstein S, Buchsbaum R, Lowenstam HA, Urey HC. Revised carbonate-water isotopic temperature scale. Geol Soc Am Bull. 1953;64:1315–1356. 10.1130/0016-7606(1953)64[1315:RCITS]2.0.CO;2

[5] Kim ST, O’Neil JR. Equilibium and nonequilibium oxygen isotope effects in synthetic carbonates. Geochim Cosmochim Acta. 1997;61:3461–3475. 10.1016/S0016-7037(97)00169-5

[6] Grossman EL, Ku TL. Oxygen and carbon isotope fractionation in biogenic aragonite: temperature effect. Org Geochem. 1986;37:1371–1382. 10.1016/168-9622(86)90057-6

[7] Bemis BE, Spero HJ, Bijma J, Lea DW. Reevaluation of the oxygen isotopic composition of planktonic foraminifera: experimental results and revised paleotemperature equations. Paleoceanography. 1998;13:150–160.

[8] Emiliani C. Pleistocene temperatures. J. Geol. 1955;63:538–78.

[9] Shackleton N. Oxygen isotope analyses and Pleistocene temperatures re-assessed. Nature. 1967;215:15–17. 10.1038/215015a01967

[10] Linsley BK, Wellington GM, Schrag DP, Ren L, Salinger MJ, Tudhope AW. Geochemical evidence from corals for changes in the amplitude and spatial pattern of South Pacific interdecadal climate variability over the last 300 years. Clim Dyn. 2004;22:1–11. 10.1007/s00382-003-0364-y

[11] Asami R, Yamada T, Iryu Y, Quinn TM, Meyer CP, Paulay G. Interannual and decadal variability of the western Pacific sea surface condition for the years 1787–2000: Reconstruction based on stable isotope record from a Guam coral. J Geophys Res. 2005;110:C05018 10.1029/2004JC002555

[12] Calvo E, Marshall JF, Palejero C, McCulloch MT, Gagan MK, Lough JM. Interdecadal climate variability in the Coral Sea since 1708 A.D. Palaeogeogr Palaeoclimatol Palaeoecol. 2007;248:190–201. 10.1016/j.palaeo.2006.12.003

[13] Felis T, Suzuki A, Kuhnert H, Dima M, Lohmann G, Kawahata H. Subtropical coral reveals abrupt early-twentieth-century freshening in the western North Pacific Ocean. Geology. 2009;37:527–530. 10.1130/G25581A.1

[14] Gagan MK, Dunbar GB, Suzuki A. The effect of skeletal mass accumulation in *Porites* on coral Sr/Ca and δ^18^O paleothermometry. Paleoceanography. 2012;27:PA1203 10.1029/2011PA002215

[15] Schöne BR, Dunca E, Mutvei H, Norlund U. A 217-year record of summer air temperature reconstructed from freshwater pearl mussels (*M*. *margarifitera*, Sweden). Quat Sci Rev 2004;23:1803–1816. 10.1016/j.quascirev.2004.02.017

[16] Schöne BR, Freyre Castro AD, Fiebig J, Houk SD, Oschmann W, Kroncke I. Sea surface water temperatures over the period 1884–1983 reconstructed from oxygen isotope ratios of a bivalve mollusk shell (*Arctica islandica*, southern North Sea). Palaeogeogr Palaeoclimatol Palaeoecol. 2004;212:215–232. 10.1016/j.palaeo.2004.05.024

[17] Carre M, Bentaleb I, Blamart D, Ogle N, Cardenas F, Zevallos S, et al Stable isotopes and sclerochronology of the bivalve *Mesodesma donacium*: Potential application to Peruvian paleoceanographic reconstructions. Palaeogeogr Palaeoclimatol Palaeoecol. 2005;228:4–25. 10.1016/j.palaeo.2005.03.045

[18] Chauvaud L, Lorrain A, Dunbar RB, Paulet YM, Thouzeau G, Jean F, et al, Mucciarone D. Shell of the Great Scallop *Pectin maximus* as a high-frequency archive of paleoenvironmental change. Geochem Geophys Geosyst. 2005;6.8 10.1029/2004GC000890

[19] Gillikin DP, Ridder FD, Ulens H, Elskens M, Keppens E, Baeyens W, et al Assessing the reproducibility and reliability of estuarine bivalve shells (*Saxidomus giganteus*) for sea surface temperature reconstruction: Implications for paleoclimate studies. Palaeogeogr Palaeoclimatol Palaeoecol 2005;228:70–85. 10.1016/j.palaeo.2005.03.047

[20] Parkinson D, Curry GB, Cusack M, Fallick AE. Shell structure, patterns and trends of oxygen and carbon stable isotopes in modern brachiopods shells. Chem Geol. 2005;219:193–235. 10.1016/j.chemgeo.2005.02.002

[21] Cusack M, Huerta AP. Brachiopods recording seawater temperature-A matter of class or maturation? Chem Geol. 2012;334:139–143. 10.1016/j.chemgeo.2012.10.021

[22] Yamamoto K, Asami R, Iryu Y. Brachiopod taxa and shell portions reliably recording past ocean environments: Toward establishing a robust paleoceanographic proxy. Geophys Res Lett. 2011;38:L13601 10.1029/2011GL047134

[23] Takayanagi H, Asami R, Abe O, Miyajima T, Kitagawa H, Sasaki K, et al Intraspecific variations in carbon-isotope and oxygen-isotope compositions of a brachiopod *Basiliola lucida* collected off Okinawa-jima, southwestern Japan. Geochim Cosmochim Acta. 2013;115:115–136. 10.1016/j.gca.2013.03.026

[24] Takayanagi H, Asami R, Otake T, Abe O, Miyajima T, Kitagawa H, et al Quantitative analysis of intraspecific variations in the carbon and oxygen isotope compositions of the modern cool-temperate brachiopod *Terebratulina crossei*. Geochim Cosmochim Acta. 2015;170:301–320. 10.1016/j.gca.2015.08.006

[25] Lamarck JB. Histoire naturelle des animaux sans vertèbres Paris: J. B. Baillière; 1819.

[26] Rosewater J. The family Tridacnae in the Indo-Pacific. Indo-Pacific Mollusca. 1965;1:347–396.

[27] Goreau TF. On the relation of calcification to primary production in reef-building organisms In: Lenhoff HH, Loomis WF, editors. The biology of Hydra and some other coelenterates. Miami: University of Miami Press; 1961 p. 269–285.

[28] Simkiss K. Phosphates as crystal poisons of calcification. Biol Rev Cambridge Philos Soc. 1964;39:487–503. 10.1111/j.1469-185X.1964.tb01166.x 14222524

[29] Aharon P, Chappell J. Oxygen isotopes, sea level changes and temperature history of a coral reef environment in New Guinea over the last 105 years. Palaeogeogr Palaeoclimatol Palaeoecol. 1986;56:337–379. 10.1016/0031-0182(86)90101-X

[30] Watanabe T, Oba T. Daily reconstruction of water temperature from oxygen isotopic ratios of a modern *Tridacna* shell using freezing microtome sampling technique. J Geophys Res. 1999;104:20667–20674. 10.1029/1999JC900097

[31] Sano Y, Kobayashi S, Shirai K, Takahata N, Matsumoto K, Watanabe T, et al Past daily light cycle recorded in the strontium/calcium ratios of giant clam shells. Nature Communications. 2012;3:761 10.1038/ncomms1763 22453834

[32] Aharon P. Recorders of reef environmental histries: stable isotopes in corals, giant clams, and calcareous algae. Coral Reefs. 1991;10:71–90. 10.1007/BF00571826

[33] Romanek CS, Grossman EL. Stable isotope profiles of *Tridacna maxima* as environmental indicators. Palaios. 1989;4:402–413. 10.2307/3514585

[34] Patzöld J, Heinrichs JP, Wolschendorf K, Wefer G. Correlation of stable oxygen isotope temperature record with light attenuation profiles in reef-dwelling *Tridacna* shells. Coral Reefs. 1991;10:65–69. 10.1007/BF00571825

[35] Elliot M, Welsh K, Chilcott C, McCulloch M, Chappell J, Ayling B. Profiles of trace elements and stable isotopes derived from giant long-lived *Tridacna gigas* bivalves: potential applications in paleoclimate studies. Palaeogeogr Palaeoclimatol Palaeoecol. 2009;280:132–142. 10.1016/j.palaeo.2009.06.007

[36] Welsh K, Elliot M, Tudhope A, Ayling B, Chappel J. Giant bivalves (*Tridacna gigas*) as recorders of ENSO variability. Earth Planet Sci Lett. 2011;307:266–270. 10.1016/j.epsl.2011.05.032

[37] Yan H, Shao D, Wang Y, Sun L. Sr/Ca profile of long-lived *Tridacna gigas* bivalves from South China Sea: A new high-resolution SST proxy. Geochim Cosmochim Acta. 2013;112:52–65. 10.1016/j.gca.2013.03.007

[38] Asami R, Konishi M, Tanaka K, Uemura R, Furukawa M, Shinjo R. Late Holocene coral reef environment recorded in Tridacnidae shells from archaeological sites in Okinawa-jima, subtropical southwestern Japan. Island Arc. 2015;24:61–72. 10.1111/iar.12076

[39] Röding PF. Museum Boltenianum sive catalogus cimeliorum e tribus regnis naturæ quæ olim collegerat Joa. Fried Bolten, M. D. p. d. per XL. annos proto physicus Hamburgensis Pars secunda continens conchylia sive testacea univalvia, bivalvia & multivalvia. Hamburg: Typis Johan. Christi. Trappii; 1798.

[40] Amante C, Eakins BW. ETOPO1 1 Arc-minute global relief model: procedures, data sources and analysis. NOAA Technical Memorandum NESDIS NGDC-24. Washington, DC.: National Oceanic and Atmospheric Administration (NOAA); 2009.

[41] Abe O, Agata S, Morimoto M, Abe M, Yoshimura K, Hiyama T, et al A 6.5-year continuous record of sea surface salinity and seawater isotopic composition at Harbour of Ishigaki Island, southwest Japan. Isotopes Environ Health Stud. 2009;45(3):247–258. 10.1080/10256010903083847 20183236

[42] Reynolds RW, Rayner NA, Smith TM, Stokes DC, Wang W. An improved in situ and satellite SST analysis for climate. J. Clim. 2002;15:1609–1625. 10.1175/1520-0442(2002)015<1609:AIISAS>2.0.CO;2

[43] Bruguière JG. Tableau encyclopédique et méthodique des trois règnes de la nature Dix-neuvième partie: Vers testacées à coquilles bivalves. Paris: Henri Agasse, 1797.

[44] Huelsken T, Keyse J, Liggins L, Penny S, Treml EA, Riginos C. A novel widespread cryptic species and phylogeographic patterns within several giant clam species (Cardiidae: *Tridacna*) from the Indo-Pacific Ocean. PLoS ONE. 2013;8:e80858 10.1371/journal.pone.0080858 24278333 PMC3835327

[45] Fankobner PV. Self righting by tridacnid clams. Nature. 1971;230:579–580. 10.1038/230579a0

[46] Okai T, Suzuki A, Terashima S, Inoue M, Nohara M, Kawahata H, et al Collaborative analysis of GST/AIST geochemical reference materials JCp-1 (Coral) and JCt-1 (Giant Clam). Chikyukagaku (Geochemistry). 2004;38:281–286.

[47] Sokal RR, Rohlf FJ. Biometry: The principal and practice of statistics in biological research 3rd ed. New York: WH, Freeman; 1994.

[48] Paillard D, Labeyrie L, Yiou P. Macintosh program makes time-series analysis easy. EOS (Washington DC). 1996;77:379.

[49] Grossman EL. Carbon isotopic fractionation in live benthic foraminifera-comparison with inorganic precipitate study. Geochim Cosmochim Acta. 1984;48:1505–1512. 10.1016/0016-7037(84)90406-X

[50] Romanek CS, Grossman EL, Jones DS. Carbon isotopic fractionation in synthetic aragonite and calcite: Effect of temperature and precipitation rate. Geochim Cosmochim Acta. 1992;56:419–430. 10.1016/0016-7037(92)90142-6

[51] Zhang J, Quay PD, Wilbur DO. Carbon isotope fractionation during gas-water exchange and dissolution of CO_2_. Geochim Cosmochimi Acta. 1995;59:107–114. 10.1016/0016-7037(95)91550-D

[52] Böhm F, Joachimski MM, Dullo WC, Eisenhauer A, Lehnert H, Reitner J, et al Oxygen isotope fraction in marine aragonite of coralline sponges. Geochim Cosmochim Acta. 2000;64(10):1695–1703.

[53] Jones DS, Williams DF, Romanek CS. Life history of symbiont-bearing giant clams from stable isotope profiles. Science. 1986;231:46–48. 10.1126/science.231.4733.46 17819230

[54] Romanek CS, Jones DS, Williams DF, Krantz DE, Radtke R. Stable isotopic investigation of physiological and environmental changes recorded in shell carbonate from the giant clam *Tridacna maxima*. Mar Biol. 1987;94:385–393. 10.1007/BF00428244

[55] Betenburg SJ, Reichart GJ, Jilbert T, Janse M, Wesselingh FP, Renema W. Interannual climate variability in the Miocene: High resolution trace element and stable isotope ratios in giant clams. Palaeogeogr Palaeoclimatol Palaeoecol. 2011;306:75–81. 10.1016/j.palaeo.2011.03.031

[56] McConnaughey TA, Gillikin DP. Carbon isotopes in mollusk shell carbonates. Geo-Mar Lett. 2008;28:287–299. 10.1007/s00367-008-0116-4

[57] Schöne BR, Wanamaker AD Jr, Fiebig J, Thébault J, Kreutz KJ. Annually resolved δ^13^C_shell_ chronologies of long-lived bivalve mollusks (*Arctica islandica*) reveal oceanic carbon dynamics in the temperate North Atlantic during recent centuries. Palaeogeogr Palaeoclimatol Palaeoecol. 2011;302:31–42. 10.1016/j.palaeo.2010.02.002

[58] Kennedy H, Richardson CA, Duarte CM, Kennedy DP. Oxygen and carbon stable isotopic profiles of the fan mussel, *Pinna nobilis*, and reconstruction of sea surface temperatures in the Mediterranean. Mar Biol. 2001;139:1115–1124. 10.1007/s002270100673

[59] Keller N, Del Piero D, Longinelli A. Isotopic composition, growth rates and biological behavior of *Chamelea gallina* and *Callista chione* from the Gulf of Trieste (Italy). Mar Biol. 2002;140:9–15. 10.1007/s002270100660

[60] Lorrain A, Paulet YM, Chauvaud L, Dunbar R, Mucciarone D, Fontugne M. δ^13^C variation in scallop shells: Increasing metabolic carbon contribution with body size? Geochim Cosmochim Acta. 2004;68:3509–3519. 10.1016/j.gca.2004.01.025

[61] Guilkin DP, Lorrain A, Bouillon S, Willenz P, Dehairs F. Stable carbon isotopic composition of *Mytilus edulis* shells: relation to metabolism, salinity, δ^13^C_DIC_ and phytoplankton. Org Geochem. 2006;37:1371–1382. 10.1016/j.orggeochem.2006.03.008

[62] Vander Putten E, Dehairs F, Keppens E, Baeyens W. High resolution distribution of trace elements in the calcite shell layer of modern *Mytilus edulis*: environmental and biological controls. Geochim Cosmochim Acta. 2000;64:997–1011. 10.1016/S0016-7037(99)00380-4

[63] Schöne BR. *Arctica islandica* (Bivalvia): A unique paleoenvironmental archive of the northern North Atlantic Ocean. Glob Planet Change. 2013;111,199–225. 10.1016/j.gloplacha.2013.09.013

[64] McConnaughey TA, Burdett J, Whelan JF, Paull CK. Carbon isotopes in biological carbonates: Respiration and photosynthesis. Geochim Cosmochim Acta. 1997;61:611–622. 10.1016/S0016-7037(96)00361-4

[65] Rollion-Bard C, Blamart D, Cuif JP, Juillet-Leclerc A. Microanalysis of C andoi:sotopes of azooxanthellate and zooxanthellate corals by ion microprobe. Coral Reefs. 2003;22(4):405–415. 10.1007/s00338-003-0347-9

[66] Hean RL, Cacho OJ. A growth model for giant clams *Tridacna crocea* and *T*. *derasa*. Ecol. Modell. 2003;163:87–100. 10.1016/S0304-3800(02)00400-3

[67] Yamamoto K, Asami R, Iryu Y. Carbon and oxygen isotopic compositions of modern brachiopod shells from warm-temperature shelf environment, Sagami Bay, central Japan. Palaeogeogr Palaeoclimatol Palaeoecol. 2010;291:348–359. 10.1016/j.palaeo.2010.03.006

[68] Yamamoto K, Asami R, Iryu Y. Within-shell variations in carbon and oxygen isotope compositions of two modern brachiopods from a subtropical shelf environment off Amami-o-sima, southwestern Japan. Geochem Geophys Geosyst. 2010;11:Q10009 10.1029/2010GC0033190

[69] Kitamura A, Tada K, Sakai S, Yamamoto N, Ubukata T, Miyaji T, et al 2011 Age and growth of *Glossocardia obesa*, a “large” bivalve in a submarine cave within a coral reef, as revealed by oxygen isotope analysis. The Veliger. 2011;51:59–65.

[70] Auclair AC, Joachimski MM, Lecuyer C. Deciphering kinetic, metabolic and environmental controls on stable isotope fractionations between seawater and the shell of *Terebratalia transversa* (Brachiopoda). Chem Geol. 2003;202:59–78. 10.1016/S0009-2541(03)00233-X

[71] Brand U, Logan A, Hiller N, Richardson J. Geochemistry of modern brachiopods: applications and implications for oceanography and paleoceanography. Chem Geol. 2003;198:305–334. 10.1016/S0009-2541(03)00032-9

[72] Yamamoto K, Asami R, Iryu Y. Correlative relationships between carbon- and oxygen-isotope records in two cool-temperate brachiopod species off Otsuchi Bay, northeastern Japan. Paleontol Res. 2013;17(1):12–26. 10.2517/1342-8144-17.1.12

[73] Takayanagi H, Asami R, Abe O, Kitagawa H, Miyajima T, Iryu Y. Carbon- and oxygen-isotope compositions of a modern deep-water brachiopod *Campagea japonica* collected off Aguni-jima, Central Ryukyu Islands, southwestern Japan. Geochem J. 2012;46:77–87. 10.2342/geochemj.1.0153

[74] Watanabe T, Suzuki A, Kawahata H, Kan H, Ogawa S. A 60-year isotopic record from a mid-Holocene fossil giant clam (*Tridacna gigas*) in the Ryukyu Islands: physiological and paleoclimatic implications. Palaeogeogr Palaeoclimatol Palaeoecol. 2004;212:343–354. 10.1016/j.palaeo.2004.07.001

[75] Richardson CA, Kennedy H, Duarte CM, Kennedy DP, Proud SV. Age and growth of the fan mussel *Pinna nobilis* from south-east Spanish Mediterranean seagrass *(Posidonia oceanica*) meadows. Mar Biol. 1999;133:205–212. 10.1007/002270050459

